# Coiled-Coil Motifs of RNA-Binding Proteins: Dynamicity in RNA Regulation

**DOI:** 10.3389/fcell.2020.607947

**Published:** 2020-11-19

**Authors:** Lenzie K. Ford, Luana Fioriti

**Affiliations:** ^1^Department of Neuroscience, Zuckerman Institute, Columbia University, New York, NY, United States; ^2^Laboratory of Molecular Mechanisms of Polyglutamine Disorders, Department of Neuroscience, Dulbecco Telethon Institute, Istituto di Ricerche Farmacologiche Mario Negri (IRCCS), Milan, Italy

**Keywords:** coiled coil, RNA binding protein, membraneless organelle, liquid liquid phase separation, neurons, amyloid, neurodegeneration

## Abstract

Neuronal granules are biomolecular condensates that concentrate high quantities of RNAs and RNA-related proteins within neurons. These dense packets of information are trafficked from the soma to distal sites rich in polysomes, where local protein synthesis can occur. Movement of neuronal granules to distal sites, and local protein synthesis, play a critical role in synaptic plasticity. The formation of neuronal granules is intriguing; these granules lack a membrane and instead phase separate due to protein and RNA interactions. Low complexity motifs and RNA binding domains are highly prevalent in these proteins. Here, we introduce the role that coiled-coil motifs play in neuronal granule proteins, and investigate the structure-function relationship of coiled-coil proteins in RNA regulation. Interestingly, low complexity domains and coiled-coil motifs are highly dynamic, allowing for increased functional response to environmental influences. Finally, biomolecular condensates have been suggested to drive the formation of toxic, neurodegenerative proteins such as TDP-43 and tau. Here, we review the conversion of coiled-coil motifs to amyloid structures, and speculate a role that neuronal granules play in coiled-coil to amyloid conversions of neurodegenerative proteins.

## Introduction

Coiled-coil motifs are abundant in RNA-binding proteins (RBP). These proteins play physiological roles in the synaptic plasticity of neurobiology. Many coiled-coil enriched RBPs are localized to liquid-liquid phase separated (LLPS) neuronal granules, a compartmentalization that is critical for appropriate RNA trafficking in local protein synthesis. However, these LLPS organelles are highly implicated in the misfolding and amyloid formation of various neurodegenerative disease-related proteins. In this perspective, we hypothesize that coiled-coil motifs within RBPs may lead to disastrous protein misfolding and neurodegenerative amyloid formation within LLPS granules.

## Coiled-Coil Motifs

**FIGURE 1:**
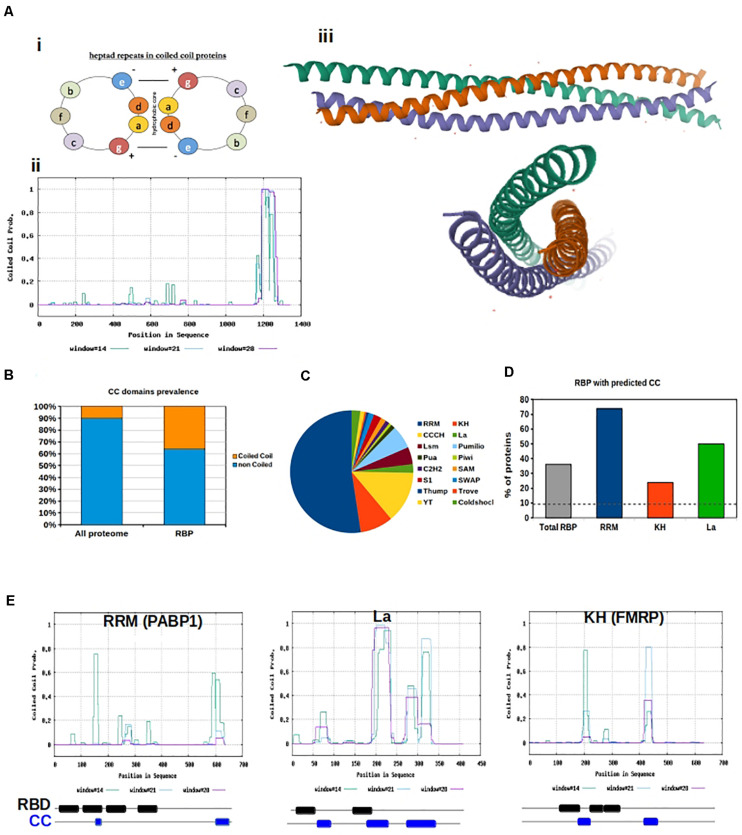
Coiled coils motifs in RNA Binding proteins. **(A) (i)** Helical wheel projection showing the position of amino acids in heptad repeats. **(ii)** Prediction of coiled coil motifs in SynGAP. The probability of coiled coil is plotted for each amino acid using the Software Paircoils. The three different colors correspond to the probability of assuming the coiled fold depending on the length of the amino acid sequence. **(iii)** Graphic representation of the Crystal structure of SynGAP coiled coil domain; **(B)** Prevalence of proteins with CC motifs in the eukaryotic proteome vs presence of CC in RBP. The software Coils was used to predict the presence of CC in RBP listed in the database RBPDB; **(C)** Pie chart representation of the abundance of different RNA binding domains in RBPs. RRM are the most abundant; **(D)** The graph shows the percentage of RBPs with Coiled coils motifs, based on the type of RNA binding domain; **(E)** Graphical representation of coiled coil prediction for PABP1, La and FMRP. Below each graph a schematic of the organization of the RNA binding domains (RBD, black) and CC motifs (blue) is shown. RBD and CC are justaxposed, with minimal or no overlap.

Coiled-coil motifs may have evolved as a means of addressing the need for increased functional complexity, expanding dynamic protein structural conformations without the creation of new genes. This is observed by the drastic increase in the presence of coiled-coil motifs in the prokaryotic genome to the 10% found in the eukaryotic genome (Rose et al., 2005). Through the supercoiling of two or more α-helices, coiled-coils form covalently bound strong supersecondary structures, as observed in the synaptic protein SynGAP (Figure 1A; PDB ID: 5JXC; Zeng et al., 2016; Sehnal et al., 2018; RCSB PDB). This motif allows for a variety of energetically feasible oligomerization and quaternary structure formation (Yadav et al., 2006). Complexity is further increased by the addition of non-covalently associated subunits, expanding the extent of protein function via increased possibility for quaternary structures (Yadav et al., 2006). Often, coiled-coil motifs function as molecular spacers between functional domains or as scaffolding for macromolecules, but have also been found to play catalytic roles in some proteins (Dong et al., 2007).

The coiled-coil motif is a well-defined heptad comprising of amino acids “a,” “b,” “c,” “d,” “e,” “f,” and “g,” where “a” and “d” are typically hydrophobic and “b,” “c,” and “f” are often charged. The hydrophobicity of amino acids “a” and “d” allow for the “knobs and holes” hydrophobic core that bonds the two or more α-helices (Truebestein and Leonard, 2016). The canonical coiled-coil has, on average, 3.5 amino acids per turn periodicity which is close to that of a standard α-helix (Truebestein and Leonard, 2016). However, non-canonical, hetero-oligomeric, and discontinuous coiled-coils exist in nature, due to variability in hydrophobic and polar residues at the third and/or fourth position as well as insertion of one, three, or four residues within the motif (Truebestein and Leonard, 2016). These discontinuities result in the formation of skips (ab**b**cdefg), stammers (abc**abc**defg), stutters (abcd**abcd**efg), and α-β coiled-coils (abcβββefg) (Truebestein and Leonard, 2016).

## Coiled-Coil Domains Are Over-Represented in RNA-Binding Proteins

Coiled-coil motifs are possibly best known for their role in eukaryotic motor protein function and cytoskeletal polymerization. However, coiled-coil motifs are prevalent in vesicle-associated/trafficked proteins as well (Rose et al., 2005). Specifically, we find that coiled-coil motifs are over-represented in RBPs, which are trafficked to sites of local protein synthesis depending on the needs of the cell. This coiled-coil interaction allows for movement of cargo proteins, lipids, and RNAs within membrane-bound and membraneless organelles via motor protein binding, along the microtubule cytoskeletal network. For example, the microtubule-associated kinesin-1 cargo adaptor complex (KLC1) moves ribonucleoprotein granules by interacting with the RBP SFPQ via a coiled-coil motif, an interaction that is necessary for axonal transport (Fukuda et al., 2020). Large, membrane-bounded organelles, such as the lysosome, can be trafficked throughout the cell via similar coiled-coil protein-protein interactions. Annexin A11 adaptor protein, a component of ribonucleoprotein granules containing mRNAs necessary for growth cone morphology, is transported throughout the neuron by “hitchhiking” on the lysosome cargo (Liao et al., 2019). The lysosome cargo can bind dynein and traffic through the cell via the microtubule network, relying heavily on coiled-coil proteins for recruitment of cargo and activation of motility (Reck-Peterson et al., 2019).

We downloaded all human RBPs listed in the RNA-binding protein database RBPDP (Cook et al., 2011) and predicted the presence of coiled-coil motifs within their sequences. We used the software Coils (Lupas et al., 1991), and confirmed the results with PCoils (McDonnell et al., 2006) and DeepCoils (Ludwiczak et al., 2019). Overall, we found a significant over-representation of RBP containing coiled-coils motifs with respect to what is expected by chance (*P* < 0.001, χ^2^ test). In fact 36% of all RBPs have coiled-coil motifs in their sequence, which is significantly higher than the 10% reference value for the total eukaryotic genome (Rose et al., 2005; Figure 1B). Since distinct RBPs have different sequences, we examined whether specific RNA-binding domains were preferentially associated with coiled-coil motifs. Domains were defined as: RNA-binding domain [RBD, also known as ribonucleoprotein domain (RNP) and RNA recognition motif (RRM)], K-homology (KH) domain (type I and type II), Arg-Gly-Gly (RGG) box, Sm domain, DEAD/DEAH box, zinc finger (ZnF), double stranded RNA-binding domain (dsRBD), ColdShock domain; Pumilio/FBF (PUF or Pum-HD) domain, and Piwi/Argonaute/Zwille (PAZ) (Figure 1C).

Variability in binding domains allows for more sophisticated nucleic acid recognition and binding. Complexity is often increased by the presence of multiple modular units within one protein. The actual binding between RBPs and RNA is the result of weak interactions, and an increase in binding domains allows the weak interaction surface to be largely increased. As a result, RBPs containing several domains can bind RNA with higher specificity and affinity than a single domain. Additionally, conformational changes within proteins or RNA can increase the binding strength We hypothesize that flexible coiled-coil motifs can allow increased access of RNA-binding domains to RNA targets. Additionally, oligomerization of coiled-coil motifs allows for more RNA-binding domains spatially. We analyzed the different classes of human RBPs included in the RBPDB to calculate their relative abundance and the presence of a coiled-coil motif. We found that all categories of RBPs, with the exception of those with Pumilio and ColdShock domains, contained a value of coiled-coil motifs well above 10% (Figure 1D and Supplementary Figure 1A).

RBPs containing RRM, La, and KH domains are particularly relevant in neurobiological function (Dreyfuss et al., 1988; Burd and Dreyfuss, 1994; Musco et al., 1996; Intine et al., 2003; Alfano et al., 2004), thus we further analyzed the sequences of RBPs containing these three domains to determine their spatial organization with respect to coiled-coil motifs (Figure 1E). First, the RRM is the most abundant domain found in RBPs. This domain is 90 amino acids in length and consists of a four-stranded β-sheet packed against two α-helices (Rebagliati, 1989; Milburn et al., 1990). Inside the RRM there are two highly conserved regions. The first is a hydrophobic segment of six residues (which is called the RNP-2 motif) and the second is an octapeptide motif (which is called RNP-1 or RNP-CS) (Ayane et al., 1991). The RRM domain is found in proteins implicated in regulation of alternative splicing, mRNA transport, and translation (Dreyfuss et al., 1988). Second, the KH domain was the first RNA-binding domain identified in human RBPs. It binds to both ssDNA and ssRNA, and is about 70 amino acids in length. The important signature sequence of this domain is (I/L/V)IGXXGXX(I/L/V). KH domains are found in a wide variety of proteins, including ribosomal proteins, transcription factors, and post-transcriptional modifiers of mRNA (Burd and Dreyfuss, 1994; Musco et al., 1996). Finally, the La protein is a 47 kDa polypeptide that often acts as an autoantigen in systemic Lupus Erythematosus and Sjogren’s Syndrome patients. It occurs in both the nucleus and the cytoplasm, where it takes on different roles. In the nucleus, La facilitates the production of tRNAs assisting in their folding and maturation (Intine et al., 2003; Alfano et al., 2004). In the cytoplasm, La facilitates the translation of specific mRNAs, protects them from endonuclease digestion, and organizes their export from the nucleus (Intine et al., 2003; Alfano et al., 2004).

Interestingly we observed that coiled-coil motifs and these RNA binding domains are often in close proximity but never overlapping (Figure 1E), confirming our hypothesis that flexible coiled-coil motifs might allow increased access of RNA-binding domains to RNA targets.

## RNA-Binding Proteins That Contain Coiled-Coil Motifs Are Over-Represented in LLPS Organelles

The structural flexibility of RBPs mandates tight cellular regulation to ensure functional structures are formed and misfolding doesn’t occur. One way in which the cell regulates structural flexibility is by localizing these proteins to LLPS organelles. LLPS organelles lack a lipid membrane, instead separating from their surrounding environment due to liquid-liquid phase separation, a biophysical phenomena that makes the organelle more dense than its environment.

**FIGURE 2:**
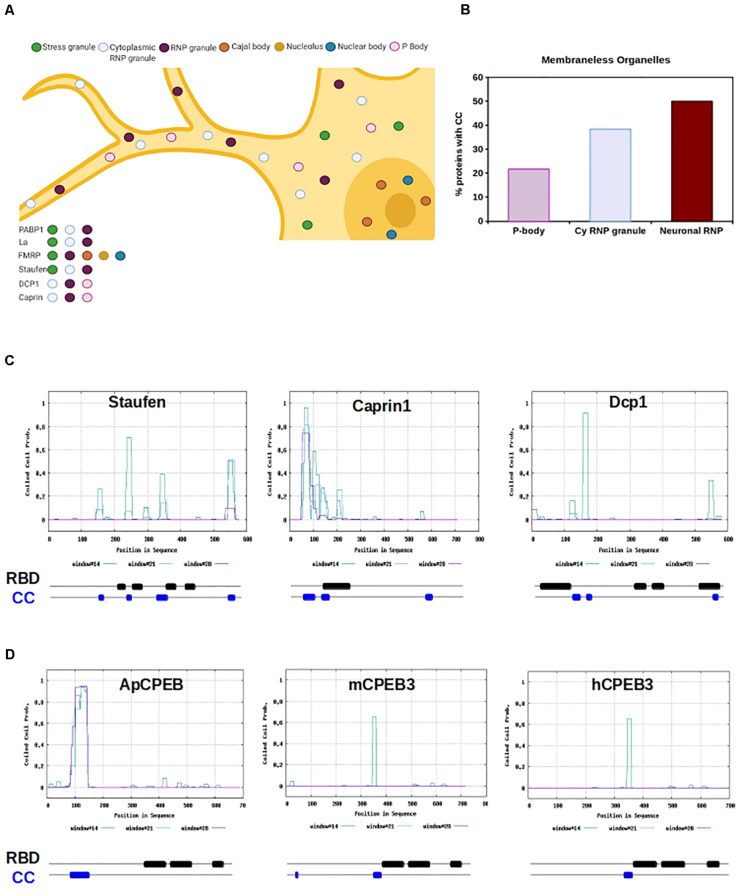
RNA Binding Proteins with CC motifs in Membraneless organelles. **(A)** Graphic representation of a neuron containing several different types of molecular condensate, within the nucleus and cytosol. RBPs can shuttle form nucleus and cytosol and move from organelles depending on the cell physiological state. Example RBPs are listed, and organelle localization is color-coded. **(B)** The chart shows the percentage of proteins with cc motifs in RBP, based on the type of organelle. P-body, cytoplasmic RNP granule (cy RNP granule), and neuronal RNP are shown. **(C)** Graphical representation of CC prediction for Staufen, Caprin1, and Dcp1. Below each graph is a schematic of the organization of the RNA binding domains (RBD, black) and CC motifs (blue) is shown. RBD and CC are justaxposed, with minimal or no overlap. **(D)** Graphical representation of CC prediction for Aplysia CPEB, mouse CPEB and human CPEB. Below each graph a schematic of the organization of the RNA binding domains (RBD, black) and CC motifs (blue) is shown. RBD and CC are justaxposed, with minimal or no overlap.

Since coiled-coils are overrepresented in RBP, we measured the prevalence of coiled-coils across various LLPS organelles, including processing bodies (P bodies), stress granules (SGs), Chromatoid Bodies (CHRBs), RNP granules, cytoplasmic RNA granules, P granules, Pi-bodies, Piwi-containing P granules (PiP)-bodies, neuronal RNP granules, and nuclear SGs (Figures 2A,B and Supplementary Figure 2). Of the organelles investigated, neuronal RNP granules contained the highest prevalence of coiled-coils, with 50% of neuronal RNPs containing a coiled-coil motif (Figure 2B).

Perhaps, coiled-coil prevalence within LLPS organelles provides structure or molecular spacing for the protein components of the organelle “core.” Additionally, the non-canonical non-covalent properties of coiled-coils may play a role in the weak interactions that characterize the organelle “shell.” These hypotheses are in line with the LLPS theory that suggests the immiscible organelle is composed of at least two layers, a core and a shell, which have distinct biophysical properties (Feric et al., 2016; Peng and Wang, 2020). Cellular application of these hypotheses are complicated by the lack of precise characterization of neuronal granules, due to their dynamicity and ability to interact and fuse with other cytoplasmic LLPS organelles (Buchan, 2014). However, P bodies, and Staufen-containing and fragile X mental retardation protein (FMRP)-containing neuronal granules are well-defined.

Processing bodies play a role in translation repression through mRNA storage (Brengues et al., 2005; Bhattacharyya et al., 2006), microRNA-mediated repression (Liu et al., 2005; Pillai et al., 2005; Teixeira and Parker, 2007), and degradation of mRNA via non-sense mediated- and normal decay (Unterholzner and Izaurralde, 2004; Sheth and Parker, 2006). These functions are ubiquitous in eukaryotic cells. Canonical P body components are abundant with predicted coiled-coil motifs, as observed in decapping protein 1 (DCP1) (NP_060873.4; nearly 100% prediction), GW182 (NP_055309.2; 100% prediction), and Piwi 2 (NP_060538.2; 40% prediction).

Neuronal P bodies have additional functions which include the transport, modification, and translation of mRNAs (Barbee et al., 2006; Kiebler and Bassell, 2006), being functionally and structurally similar to the well-characterized neuronal granules that contain Staufen and FMRP (Barbee et al., 2006). Of neuronal granules, one of the best understood is the double-stranded RBP Staufen-containing neuronal granules, which traffic mRNAs into dendrites via microtubules (Kohrmann et al., 1999; Tang et al., 2001; Figures 2A,C). The predicted presence of coiled-coils in Staufen is high (NP_059348.2; ∼70%); functionally, Staufen RBD binds coiled-coil regions of interactors in neuroblast development (Yousef et al., 2008; Jia et al., 2015). Furthermore, while Staufen remains within its neuronal granule after stimulation, the non-canonical coiled-coil protein Caprin-1 leaves the neuronal granule when localized to the dendritic ribosome after BDNF stimulation, allowing for translation of its mRNA targets under specific physiological conditions (Shiina et al., 2005). It appears that the coiled-coil motif in Caprin-1 is both necessary for neuronal granule formation, and is sufficient for its major RNA-binding ability (Shiina et al., 2005; Figures 2A,C).

The movement of synaptic transcripts to specific distal sites is necessary for growth, synaptic plasticity, and the strengthening of neuronal connections. This requires inhibition of translation until the transcript is appropriately localized, a process in which coiled-coils play an important functional role (Shiina et al., 2005; Fiumara et al., 2010; Figure 2C). Several proteins are critically implicated in this phenomenon, among which the interplay between the Polyadenylate binding protein (PABP) (Kahvejian et al., 2005) and initiations factors is particularly relevant (Bernstein et al., 1989; Burd et al., 1991; Chang et al., 2004; Kozak, 2006; Bag and Bhattacharjee, 2010). This fine-tuned regulation is a critical component of greater cognitive processes such as long-term memory (Richter and Lorenz, 2002; Klann and Dever, 2004). Beside PABP, other well studied RBPs in this context are FMRP and CPEB.

Fragile X mental retardation protein is localized to the synapse upon metabotropic glutamate receptor activation, where it functions to target dendritic mRNAs and regulates translation under specific physiological conditions (Jin and Warren, 2003; Antar et al., 2004; Figure 2A). FMRP, as well as paralogs FXR1 and FXR2, are known to homo- and hetero-dimerize via coiled-coil motifs, although the functional consequences of this are unknown (Winograd and Ceman, 2011; Figure 1D). Lack of FMRP produces Fragile X Mental Retardation in humans, and in the mouse model of the disease, neural spine morphology is disrupted and forms excessively long and thin filopodia-like structures (Nimchinsky et al., 2001). The spine morphology of this mouse model is predicated to be a direct result of FMRP disruption, which likely has widespread consequences on synaptic plasticity, and learning and memory (Klann and Dever, 2004).

Within both neuronal P bodies and FMRP-containing neuronal granules, α*CamKII* is bound and trafficked via the cytoplasmic polyadenylation element (CPE) in its 3’ untranslated region (UTR) by the RBP cytoplasmic polyadenylation element binding protein 1 and 3 (CPEB1, CPEB3) (Huang et al., 2003; Ford et al., 2019, 2020). Many dendrite-bound mRNAs contain CPE elements and neuronal granules contain a large amount of CPE element-containing mRNAs (Martin and Ephrussi, 2009). The CPE elements promote cytoplasmic polyadenylation-induced translation of the mRNAs in response to synaptic stimulation, such as NMDA-dependent long-term potentiation (Huang et al., 2002; Fioriti et al., 2015). Indeed, CPEB3 binds CPE elements of dendrite-bound mRNAs and is necessary for memory persistence (Fioriti et al., 2015). CPEB3 moves from the DCP1-containing P body to the distally located polysome after chemically induced long-term potentiation (Ford et al., 2019), and is present in FMRP-containing neuronal granules (Ford et al., 2020). CPEB3 prediction for the presence of coiled-coils is high (NP_938042.2, ∼70%) and the *Aplysia* ortholog ApCPEB has a coiled-coil motif that allows the protein to localize to neuronal, cytoplasmic LLPS organelles (Fiumara et al., 2010; Figure 2C). The role of coiled-coils in ApCPEB function was further bolstered by computational data, suggesting that low-n oligomer ApCPEB exists through coiled-coil interactions (Chen et al., 2016).

## Coiled Coil to β-Sheet Conversion

Kandel, Fioriti, and Lindquist publicized the concept of functional amyloids in the brain by their novel discoveries of functional ApCPEB and CPEB3 aggregates (Si et al., 2003; Drisaldi et al., 2015; Fioriti et al., 2015; Stephan et al., 2015). Interestingly, ApCPEB and its orthologs are RBPs with significant areas of intrinsic disorder and coiled-coil motifs (Fiumara et al., 2010; Stephan et al., 2015; Figure 2C). The notion of incremental structural conversion from intrinsic disorder to coiled-coil to β-sheet of ApCPEB was first hypothesized by Kandel and Hendrickson, and later explained through computational modeling (Fiumara et al., 2010; Chen et al., 2016). The model suggests that as concentrations of ApCPEB increase, as could occur in highly concentrated LLPS organelles (Ford et al., 2019), coiled-coil ApCPEB is poised to form β-sheet-rich structures in the upstream polyQ region (Chen et al., 2016). Remarkably, this model fits within the known mechanisms of neurodegenerative Huntingtin amyloidogenesis (Fiumara et al., 2010), spinocerebellar ataxia SCA3 amyloidogenesis (Kwon et al., 2018), and cleidocranial dysplasia RUNX2 aggregation and toxicity (Pelassa et al., 2014).

It is interesting to speculate that coiled-coil oligomerization of polyQ-rich proteins could lead to polyQ β-sheet formation, given appropriate environmental conditions. Additionally, data indicates there could be structural conversion of coiled-coils themselves to cross β-sheet amyloids. Biochemically, coiled-coils and β-sheets are facial amphiphiles; hydrophobic packing allows for strengthening of the hydrogen-bonding network, a key difference being that β-sheets have intermolecular hydrogen bonding as opposed to the coiled-coil intramolecular bonding (Dong and Hartgerink, 2007). *In vitro* and *in silico*, coiled-coil peptides can convert from an α-helical to β-sheet fibril when heated (Ciani et al., 2002; Dong and Hartgerink, 2006; Steckmann et al., 2017) or subjected to pH change (de Freitas et al., 2018). Rendering the coiled-coil structurally unstable could lead to β-sheet formation; one way in which this may occur is through a discontinuity in the heptad, creating a longer hydrophobic patch that is more favorable to β-sheet formation (Ciani et al., 2002; Dong and Hartgerink, 2007).

Furthermore, post-translational modifications may play a role in coiled-coil to amyloid fibrillization. Post-translational modifications are already heavily implicated in the aggregation or solubilization of various neurodegenerative proteins. For example, phosphorylation of amyotrophic lateral sclerosis (ALS)-related RBP fused in sarcoma (FUS) reduces the protein’s propensity to aggregate (Monahan et al., 2017). An additional ALS-related RBP, TAR-DNA binding protein 43 (TDP-43), also exhibits increased solubility and decreased aggregation when phosphorylated (Li et al., 2018). However, it is important to note that post-translational modifications have an effect on conformational change and protein aggregation in a protein-specific manner (for example, see Ford et al., 2020). Still, it is tantalizing to compare the *in vivo* amyloid studies to *in vitro* coiled-coil studies.

*In vitro* coiled-coil peptides that convert to β-sheet amyloids can be suppressed by monophosphorylation of the peptide (Broncel et al., 2010). Even when the charge of the single phosphate moiety is neutralized, β-sheet formation is inhibited (Broncel et al., 2010). Once protein phosphatase lambda is added to the solution, the peptide clearly converts from an α-helical to β-sheet state (Broncel et al., 2010). This appears to be similar to how FUS and TDP-43 are altered in regards to phosphorylation. It is critical to validate these speculations under physiological conditions, and to carefully consider differences in modifications across proteins.

## Discussion

In this perspective, we identify a high abundance of coiled-coil motifs within RBPs and discern over-representation of these motifs based on the specific RNA-binding domains present. We speculate that the possibility for flexibility and/or oligomerization allows for more dynamic functions in RBPs with specific RNA-binding domains. In fact our analysis shows that coiled-coil motifs do not overlap with RNA-binding domains, thus suggesting that they are not directly implicated in RNA binding. Instead they flank RBD, and this modular organization might allow protein-protein interactions necessary to assemble phase separated RNA granules and/or to dock them to motor proteins for their transport along neuronal projections. We then focus on functional LLPS organelles, P bodies and neuronal granules, and identify the abundance of coiled-coil motifs in LLPS organelle components. We find that, again, coiled-coil motifs are over-represented in these biophysically unique organelles. Furthermore, we link coiled-coil RBP components of P bodies and neuronal granules with functional relevance to neurobiology. Finally, we review the biochemical literature that suggests that coiled-coils may convert to amyloid β-sheets. This in turn could give rise to neurodegenerative and aggregated structures.

Critically, the *in vitro* coiled-coil peptide switching studies must be investigated within neurons. With current technology, this is an extremely difficult task. However, we believe that the study of LLPS organelles in neurobiology provides an ideal physiologically relevant approach. Functional amyloids exist in these organelles (Raveendra et al., 2013; Fioriti et al., 2015; Ford et al., 2019, 2020). Furthermore, recent neurodegenerative studies have identified the presence of amyloidogenic TDP-43, FUS, and hnRNP A/B (in Amyotrophic Lateral Sclerosis) and amyloidogenic tau (in Frontotemporal Dementia) in LLPS organelles in brain cells (Wolozin, 2012; Lenzi et al., 2015; Vanderweyde et al., 2016; Apicco et al., 2018; Khalfallah et al., 2018; Wegmann et al., 2018; Fang et al., 2019; Zhang et al., 2020). These data pose the question: do LLPS organelles play a role in amyloid formation, and if so, can we utilize this for coiled-coil to β-sheet switching studies?

Liquid-liquid phase separated organelles are highly concentrated in proteins, often with significant regions of disorder, and RNAs. *In vitro*, LLPS proteins have been observed to change states from liquid-like to gel-like and solid (Shin and Brangwynne, 2017). The proteins within these more solid structures appear to fibrilize and, in some cases, form amyloids (Lin et al., 2015; Molliex et al., 2015; Murakami et al., 2015; Patel et al., 2015). Mutations in proteins linked to neurodegenerative pathology also appear to exist within these gel-like LLPS structures (Kato et al., 2012; Kwon et al., 2013; Molliex et al., 2015; Patel et al., 2015). Much of the fibrillization and solidification has been linked to the highly concentrated environment, and the specific components of these LLPS organelles. One of the key properties of LLPS is weak, multivalent interactions between intrinsically disordered regions and/or defined modular domains (Alberti et al., 2019; Feng et al., 2019). Interestingly, multivalent electrostatic forces are predicted to play a role in α to β switching of coiled-coil peptides (Ciani et al., 2002) and this electrostatic-driven switching produces reversible amyloids *in vitro* (Ciani et al., 2002). It is thus very plausible that the LLPS environment is prime for the formation of functional and pathogenic amyloids from coiled-coil structural switching, due to molecular crowding, electrostatic forces, and other considerations unique to the LLPS environment.

Furthermore, the LLPS field is booming in the production of new technology (Tang, 2019) and universal methods of study (Alberti et al., 2019). For the past 5 years, the field has been moving from *in vitro* to *in vivo* work, expanding the physiological relevance and understanding of these studies. We believe that coiled-coil to β-sheet switching within the context of LLPS biology is a feasible means to investigate technically difficult protein biochemistry within a physiological context. If coiled-coils indeed play a role in functional or pathological amyloid formation, the production of therapeutic coiled coil peptides may provide an already established avenue for disease intervention (Woolfson, 2005).

## Bioinformatic Analysis

The FASTA protein sequences of the complete list of RBP in the RBPDB of *Homo sapiens* were downloaded from the Uniprot online database of complete reference proteomes (available at)^1^. The Paircoil2 CC-prediction software was downloaded from^2^. *Ad hoc* scripts were generated to identify the proteins containing predicted CC domains according to Paircoil2, using a *P*-score <0.05 as a detection threshold. Observed and expected values were then compared statistically using the CHI-squared test. Data were analyzed quantitatively using Excel (Microsoft). We used the Software AmiGo2 to retrieve the list of the RBP distributed in each phase separated organelle. We then extracted the protein sequences from Uniprot and run Paircoils2 to identify the proteins containing predicted CC domains.

## Data Availability Statement

Publicly available datasets were analyzed in this study. The datasets analyzed for this study can be found in the RNA Binding protein database (http://rbpdb.ccbr.utoronto.ca) and AmiGO2 (http://amigo.geneontology.org/amigo).

## Author Contributions

LF performed bioinformatic analyses. LKF and LF wrote the manuscript. Both authors contributed to the article and approved the submitted version.

## Conflict of Interest

The authors declare that the research was conducted in the absence of any commercial or financial relationships that could be construed as a potential conflict of interest.

## Footnotes

1 http://www.uniprot.org

2 http://groups.csail.mit.edu/cb/paircoil2/

## Supplementary Material

Supplementary Figure 1 Coiled coils motifs in RNA Binding proteins. **(A)** Prevalence of proteins with CC motifs in the human database of RBP, divide by type of RNA binding domain. The software Coils was used to predict the presence of CC in RBP listed in the database RBPDB; **(B)** Graphical representation of coiled coil prediction for members of each family. Below each graph a schematic of the organization of the RNA binding domains (RBD, black) and CC motifs (blue) is shown. RBD and CC are justaxposed, with minimal or no overlap.

Supplementary Figure 2 **(A)** The chart shows the percentage of proteins with cc motifs in RBP, based on the type of organelle in which they resides. Neuronal RNPs, chromatoid bodies, cytosolic RNP, and Piwi-containing P granules possess the highest percentage of components with predicted CC motifs. The Software AmiGo2 was used to download the RBP distributed in each organelle.
