# Supplementary figures and images for: Coiled-Coil Motifs of RNA-Binding Proteins: Dynamicity in RNA Regulation
Source: Front Cell Dev Biol. 2020 Nov 19;8:607947. doi: 10.3389/fcell.2020.607947 (PMC7710910; doi:10.3389/fcell.2020.607947)

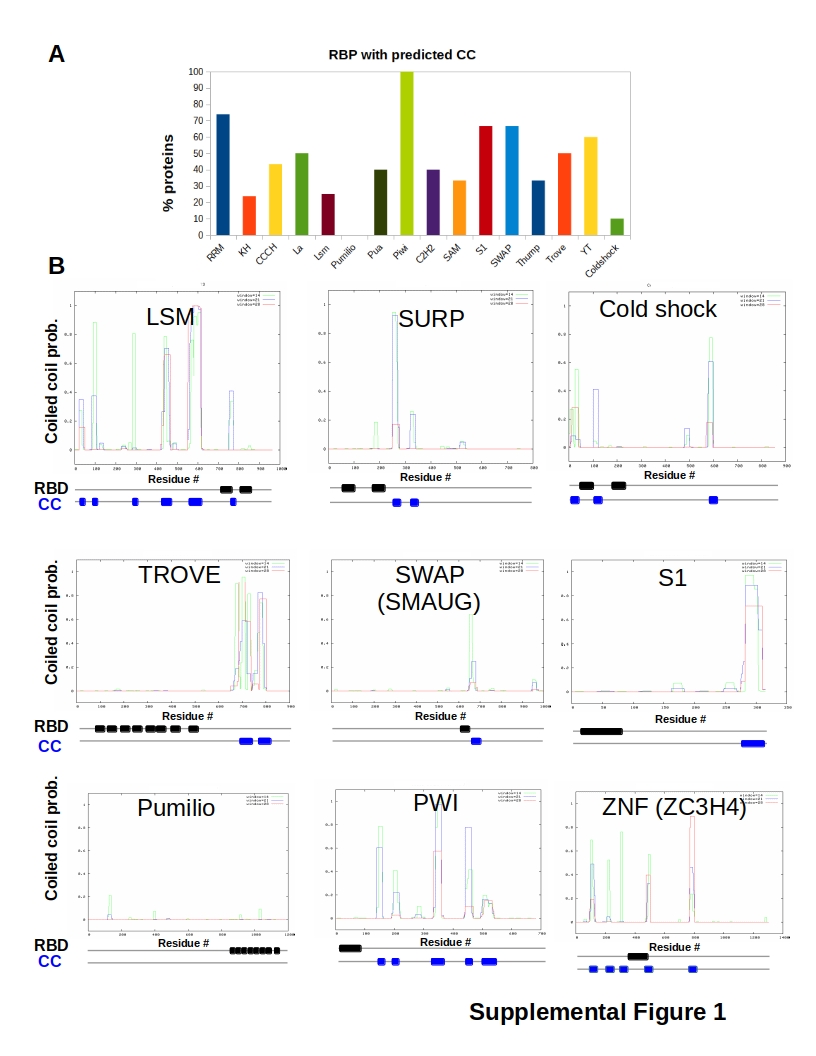

Supplement: Supplementary Figure 1 — Coiled coils motifs in RNA Binding proteins. (A) Prevalence of proteins with CC motifs in the human database of RBP, divide by type of RNA binding domain. The software Coils was used to predict the presence of CC in RBP listed in the database RBPDB; (B) Graphical representation of coiled coil prediction for members of each family. Below each graph a schematic of the organization of the RNA binding domains (RBD, black) and CC motifs (blue) is shown. RBD and CC are justaxposed, with minimal or no overlap. [file Image_1.JPEG]

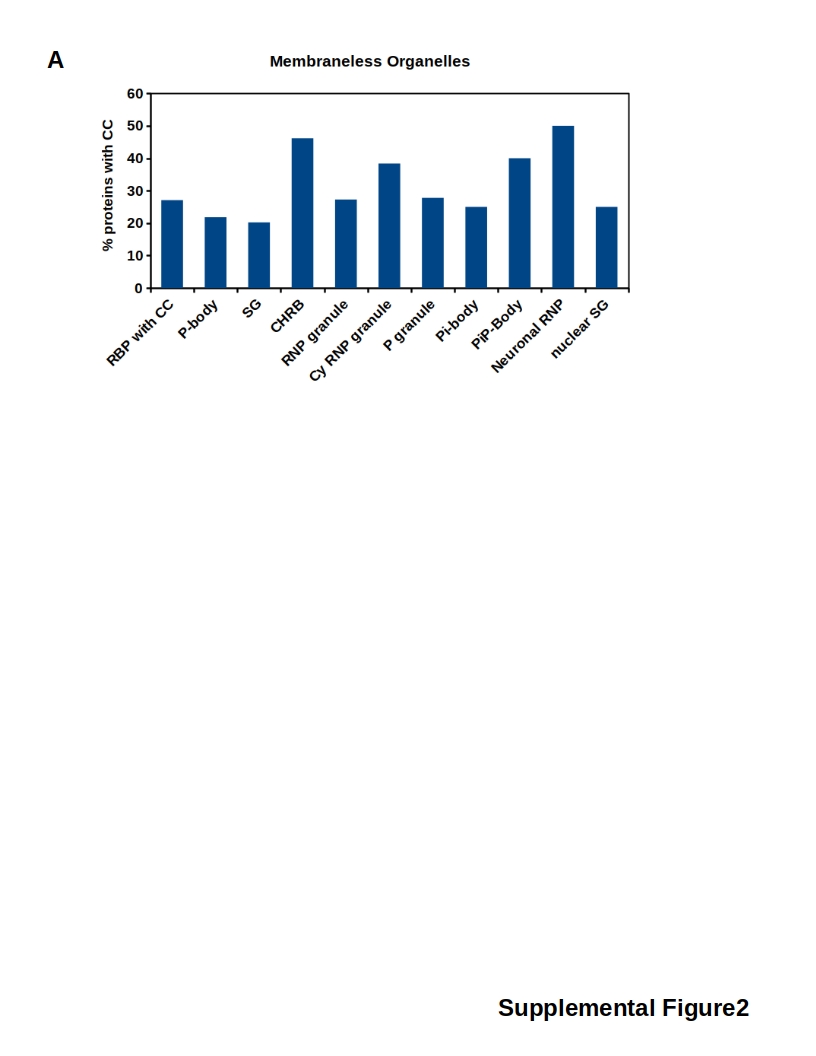

Supplement: Supplementary Figure 2 — (A) The chart shows the percentage of proteins with cc motifs in RBP, based on the type of organelle in which they resides. Neuronal RNPs, chromatoid bodies, cytosolic RNP, and Piwi-containing P granules possess the highest percentage of components with predicted CC motifs. The Software AmiGo2 was used to download the RBP distributed in each organelle. [file Image_2.JPEG]
